# Are there other undiagnosed tick-borne infections in children being evaluated for Lyme neuroborreliosis?

**DOI:** 10.1186/s12887-026-07000-4

**Published:** 2026-06-15

**Authors:** Henrik Hillerdal, Malin Lager, Anna Grankvist, Kenneth Nilsson, Christine Wennerås, Anna J Henningsson, Barbro H Skogman

**Affiliations:** 1 Department of Pediatrics, Region Jönköping County, Jönköping, Sweden; 2 Department of Biomedical and Clinical Sciences, Division of Inflammation and Infection, Linköping University, Linköping, Sweden; 3 Department of Clinical Microbiology, National Reference Laboratory for Tick-Borne Bacteria, Region Jönköping County, Jönköping, Sweden; 4 Department of Clinical Microbiology, Sahlgrenska University Hospital, Gothenburg, Sweden; 5 Department of Medical Sciences, Section of Clinical Microbiology, Uppsala University, Uppsala, Sweden; 6 Department of Infectious Diseases, Institute of Biomedicine, Sahlgrenska Academy, University of Gothenburg, Gothenburg, Sweden; 7 Center for Clinical Research Dalarna, Uppsala University, Falun, Sweden; 8 School of Medical Sciences, Örebro University, Örebro, Sweden

**Keywords:** Real-time PCR, Emerging tick-borne diseases, Pediatric, Lyme neuroborreliosis, Tick-borne pathogens

## Abstract

**Background:**

There is an increasing amount of reported clinical cases of emerging tick-borne diseases (eTBDs) such as human granulocytic anaplasmosis, neoehrlichiosis, babesiosis and rickettsiosis. The incidence of eTBDs in children is rather unknown, potentially due to low awareness regarding these diseases among health professionals. The clinical picture may be unspecific and access to laboratory tests limited. We aimed to investigate the incidence and clinical manifestations of eTBDs, including possible co-infections, within a pediatric patient group evaluated for Lyme neuroborreliosis (LNB).

**Methods:**

In a cohort of Swedish children being evaluated for LNB (*n* = 235) during 2011–2014, samples and data on clinical manifestations and laboratory findings were prospectively collected. Plasma samples were analysed by real-time polymerase chain reaction (PCR) for detection of *Anaplasma phagocytophilum*,* Neoehrlichia mikurensis*,* Babesia* species (spp.) and *Rickettsia* spp. Cerebrospinal fluid from a subset of the patients was also analysed for *Rickettsia* spp. by real-time PCR.

**Results:**

No evidence of eTBDs, nor tick-borne co-infections, was found in the analysed samples from children evaluated for LNB.

**Conclusions:**

We conclude that no evidence for potential eTBDs was found within a Swedish pediatric patient group with potential high exposure to tick-borne pathogens. However, given the indications of the emergence of ticks and several tick-borne pathogens, we suggest that eTBDs should be considered as differential diagnoses in patients with non-LNB, atypical LNB or unexplained fever and skin rashes, including a complete blood count and liver enzymes in the diagnostic workup.

**Supplementary Information:**

The online version contains supplementary material available at 10.1186/s12887-026-07000-4.

## Introduction

Tick-borne diseases, transmitted by ticks within the genus *Ixodes*, are spreading in the northern hemisphere and the incidence is predicted to increase even further in the future. There are different factors contributing to this increase, but climate changes are considered as one important part (warmer temperature, change of rain patterns which lead to a prolonged season and an expanded geographical distribution for both ticks and their hosts) [1–3]. Two of the most common and well-known tick-borne infections in Sweden are Lyme borreliosis (LB), caused by species within the *Borrelia burgdorferi* sensu lato (s.l.) species complex, and tick-borne encephalitis (TBE), caused by the TBE virus. Worldwide, many other less common tick-borne pathogens (TBP) occur [4]. Among these, pathogens such as *Anaplasma phagocytophilum*,* Neoehrlichia mikurensis*,* Babesia* spp., and *Rickettsia* spp. are detected in *Ixodes ricinus* ticks, the principal vector of human infections in Europe [5–7]. There is an increasing number of reports of clinical cases of disease with these emerging TBP in northern European countries including Sweden, which can classify them as emerging tick-borne diseases (eTBD) [4, 7–13]. Although there are a lot of potential emerging TBPs [4], in this study we only refer to diseases caused by *A. phagocytophilum*,* N. mikurensis*,* Babesia* spp. and *Rickettsia* spp. when discussing eTBD. In Sweden the first clinical reports of human disease caused by these different eTBPs have been published and presented regarding babesiosis in 1992, human cases of both anaplasmosis and rickettsiosis in 1999, whereas neoehrlichiosis was first identified in a Swedish patient in 2010 [14–17]. Most of the reported cases of eTBD consist of adult patients, often with impaired immunity, and with severe clinical presentation [18–24], but less is known about the pediatric population [25]. Studies on IgG seroprevalences show that populations in different parts of Sweden and the neighbouring Nordic countries are exposed to these TBPs [10, 11, 26–28]. Asymptomatic infections most certainly occur and could, especially in children, be one reason explaining the discrepancy between seroprevalences and reports of clinical cases. A study of patients with Babesia infection showed that 4 of 10 of children were asymptomatic, compared to 13 of 67 adults [29]. Other reasons, such as potentially missed diagnoses due to mild and unspecific symptoms, limited knowledge of the diseases and their clinical presentation, or limited access to specific laboratory tests may also contribute to this discrepancy [7, 11, 28, 30]. Although clinical symptoms, signs and laboratory findings may differ between different eTBDs, many unspecific symptoms (such as fever, headache, fatigue, malaise and myalgia) are overlapping and similar [24, 25, 31–35]. Symptomatic children with anaplasmosis tend to have more prominent abdominal symptoms as compared to adults [25]. Lyme neuroborreliosis (LNB) is the second most common manifestation of LB in both children (28%) and adults (14%) [36]. LNB in children usually presents with facial nerve palsy and/or subacute meningitis [37, 38]. Adults mainly suffer from meningo-radiculitis, meningitis, encephalitis, and myelitis [39]. However, regarding LNB, non-specific symptoms (such as headache, fatigue, fever, malaise, and nausea) have also been described in both the pediatric and adult population [37, 38, 40–43]. This, together with the fact that other eTBD can cause neurological symptoms [44–46], makes clinical decision-making challenging for the pediatrician. *Rickettsia* spp. has been of particular interest due to publications regarding its association with potential neurological illness in Sweden [47–49].

Moreover, studies show that a single tick may contain several pathogens and may potentially transmit them to humans during blood feeding. Humans may also be bitten by several ticks at the same time or during a short time period and may potentially contract several different TBP, here referred to as human co-infections [19, 50–55]. A systematic review by Boyer et al. [56] deals with this complex term “co-infections”, which includes several different entities. It may be referred to as multiple seropositivities without an associated disease (asymptomatic co-infection) or two infections, but only one of them expresses clinical symptoms (symptomatic co-infections). On the other hand, simultaneous clinical symptoms of infections by two or more TBP (co-disease) may also occur [56]. There are also studies suggesting that if the tick carries multiple pathogens, it may also cause either a cooperative or a competitive pathogen interaction within the vector or the infected host. It may modulate the immune response, facilitate or obstruct the transmission to the host, influence the clinical manifestations, and/or alter the severity of symptoms [53, 54, 57–59].

We need a better knowledge regarding the clinical importance of eTBDs, including tick-borne co-infections/co-diseases. By using real-time polymerase chain reaction (PCR) for the detection of *A. phagocytophilum*,* N. mikurensis*,* Babesia* spp., and *Rickettsia* spp. in samples from children being evaluated for LNB, we aim to investigate the prevalence of these potential eTBDs within a patient group with presumed high exposure to TBP and evaluate their clinical manifestations.

## Materials and methods

### Study design and study population

Samples were prospectively collected as part of a previous observational study of children being evaluated for LNB during 2011–2014 at seven pediatric departments in a LB endemic area in South-Central Sweden [60], with a focus on PCR as a complementary diagnostic method for detection of *B. burgdorferi* s.l. in cerebrospinal fluid (CSF). Data on clinical characteristics and laboratory findings were prospectively collected using a standardized questionnaire at inclusion and at a 2-month follow-up. All samples were drawn from patients at inclusion, before antibiotic treatment, and sent by lined envelopes to a biobank at the Department of Clinical Microbiology, Laboratory Medicine, Jönköping, and stored (without being thawed) at -70 °C until analysed. When samples were further transferred to other laboratories for analyses (the Department of Clinical Microbiology, Gothenburg 2016) or the Department of Medical Sciences, Section of Clinical Microbiology, Uppsala, 2015), they were carefully held on ice until arrival and analyses.

In this present study, patients were selected based on the availability of remaining frozen samples in the biobank; 2 mL centrifuged EDTA plasma (Material A and B) and 0,5 mL non-centrifuged CSF (Material C). As a consequence there weren´t enough available sample volume from every child to perform all analyses. Analyses were performed at different sites due to specific laboratory capabilities at the time.

All samples that were analysed derived from the total of 235 children being evaluated for LNB. Patients were classified as definite LNB (*n* = 65) and possible LNB (*n* = 33) according to diagnostic criteria established by the European Federation of Neurological Societies [61]. Patients not fulfilling these criteria were classified as non-LNB (*n* = 137). No patient had anemia, leukopenia or neutropenenia. Clinical characteristics and laboratory findings are shown in Table 1.

**Table 1:** Clinical characteristics and laboratory findings of children being evaluated for Lyme neuroborreliosis (*n* = 235)

	Definite LNB (*n* = 65)	Possible LNB (*n* = 33)	Non-LNB (*n* = 137)	All patients (*n* = 235)
Gender female, *n* (%)	28 (43.1)	14 (42.4)	84 (61.3)	126 (53.6)
Age, median (range)	6 (2–15)	8 (4–15)	13 (1–19)	10 (1–19)
Recognized tick bite, *n* (%)	40 (61.5)	16 (48.5)	57 (41.6)	113 (48.1)
Duration of neurological symptoms				
0–2 weeks, *n* (%)	50 (77)	25 (76)	50 (36.5)	125 (53.2)
3–4 weeks, *n* (%)	10 (15.4)	4 (12.1)	12 (8.8)	26 (11.1)
1–2 months, *n* (%)	1 (1.5)	0 (0)	8 (5.8)	9 (3.8)
> 2 months, *n* (%)	1 (1.5)	2 (6.1)	30 (21.9)	33 (14.0)
Not specified, *n* (%)	3 (4.6)	2 (6.1)	37 (27)	42 (17.9)
Clinical manifestations and symptoms at admission				
Facial nerve palsy, *n* (%)	44 (67.7)	24 (72.7)	42 (30.7)	110 (46.8)
Headache, *n* (%)	46 (70.8)	24 (72.7)	99 (72.3)	169 (71.9)
Fatigue, *n* (%)	59 (90.8)	23 (69.7)	92 (67.2)	174 (74.0)
Fever, *n* (%)	35 (53.8)	12 (36.4)	25 (18.2)	83 (35.3)
Neck pain, *n* (%)	34 (52.3)	17 (51.5)	36 (26.3)	87 (37.0)
Neck stiffness, *n* (%)	22 (33.8)	11 (33.3)	21 (15.3)	54 (23.0)
Loss of appetite, *n* (%)	41 (63.1)	18 (54.5)	47 (34.3)	106 (45.1)
Nausea, *n* (%)	22 (33.8)	12 (36.4)	48 (35.0)	82 (34.9)
Vertigo, *n* (%)	9 (13.8)	7 (21.2)	62 (45.3)	78 (33.2)
Other symptoms, *n* (%)	46 (70.8)	16 (48.5)	54 (39.4)	116 (49.4)
Erythema migrans, *n* (%)	28 (43.1)	9 (27.3)	18 (13.1)	55 (23.4)
Laboratory data on admission				
Pleocytosis^*^, *n* (%)	65 (100)	32 (97.0)	6^a^ (4.4)	103 (43.8)
Pleocytosis^*^, median (range)	164 (20–890)	65 (< 5-1125)	0^a^ (< 5–74)	NoA
Positive Borrelia AI^**^, *n* (%)	65 (100)	1^b^ (3.0)	0 (0)	66 (28.1)
Antibiotic treatment, *n* (%)	65 (100)	33 (100)	25 (18.2)	123 (52.3)
Complete clinical recovery at 2-months follow-up, *n* (%)	56 (86.2)	28 (84.8)	105 (76.6)	189 (80.4)

*LNB* Lyme neuroborreliosis, *AI* antibody index, *CSF* cerebrospinal fluid, *n* number, *Ig* immunoglobulin, *NoA* not available

^a^Six patients with pleocytosis, but with other diagnosis established (viral meningitis, enterovirus, encephalitis)

^b^One patient with positive Borrelia AI, but with no pleocytosis

^*^ Pleocytosis: ≥5 × 10^6^/L leukocytes in CSF (90% of total cell count)

^**^ Anti-Borrelia IgG and/or IgM antibody index (AI) > 0.3 in cerebrospinal fluid by IDEIA Lyme neuroborreliosis kit (IgM and IgG) (Oxoid, Hampshire, UK)

Material A, consisting of 193 plasma samples, was analysed by real-time PCR for *A. phagocytophilum*,* Rickettsia* spp., *N. mikurensis*, and *Babesia* spp. in 2018 at the Department of Clinical Microbiology, Laboratory Medicine, Ryhov County Hospital, Jönköping, Sweden.

Material B, consisting of 196 plasma samples, was analysed by real-time PCR for *N. mikurensis* in 2016 at the Department of Clinical Microbiology, Sahlgrenska University Hospital, Gothenburg, Sweden.

Material C, consisting of 67 CSF samples, was analysed by real-time PCR for *Rickettsia* spp. in 2015 at the Department of Medical Sciences, Section of Clinical Microbiology, Uppsala University, Uppsala, Sweden. Among these 67 CSF samples, pleocytosis (≥ 5 × 10^6^/L leukocytes in CSF) was present in 20 samples in the definitive LNB group and 10 samples in the possible LNB group.

**Fig. 1:**
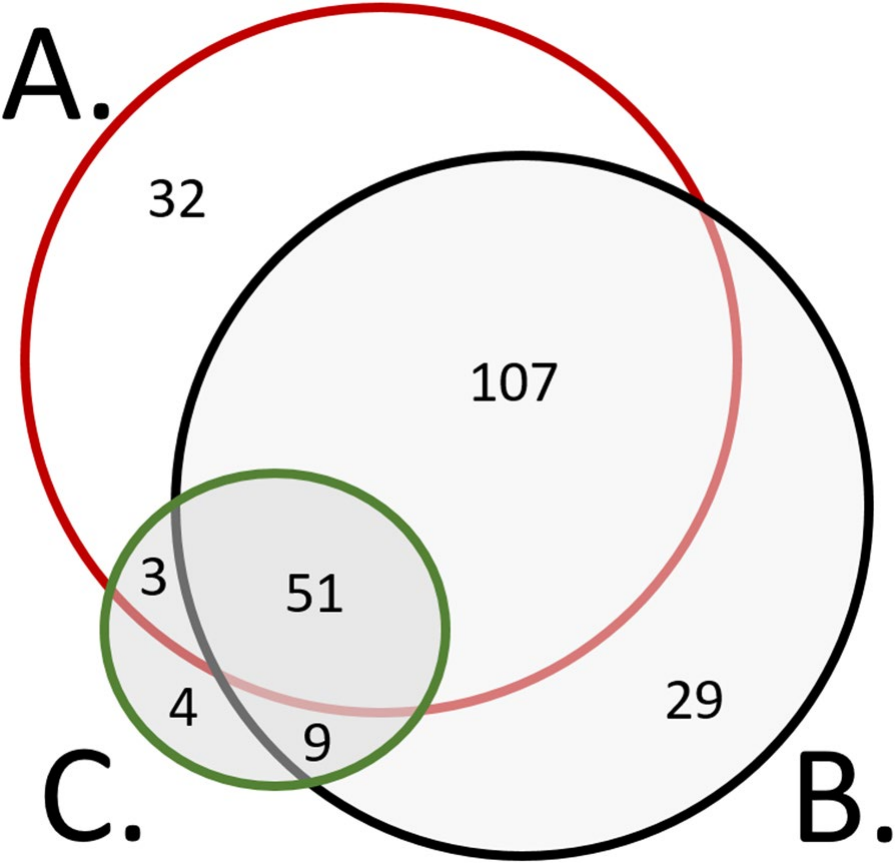
Number of samples analysed in each material from the total of 235 children. **Material** **A**. Analyses of plasma (n = 193) with real-time PCR for *A. phagocytophilum, Rickettsia* spp., *N. mikurensis*, and *Babesia* spp. in 2018 at Department of Clinical Microbiology, Laboratory Medicine, Ryhov Hospital, Jönköping, Sweden. **Material** **B**. Analyses of plasma (n = 196) with real-time PCR for *N. mikurensis* in 2016 at Department of Clinical Microbiology, Sahlgrenska University Hospital, Gothenburg, Sweden. **Material** **C**. Analyses of CSF (n = 67) with real-time PCR for *Rickettsia* spp. in 2015 at Department of Medical Sciences, Section of Clinical Microbiology, Uppsala University, Uppsala, Sweden

The distribution of children, samples, LNB classification and performed analyses within materials A-C is shown in Table 2; Fig. 1.

**Table 2:** Number of patients in material A-C in relation to Lyme neuroborreliosis classification

	Definite LNB (*n* = 65 )	Possible LNB (*n* = 33)	Non LNB (*n* = 137)	Total (*n* = 235)
Material A, patients, *n* (%)	63 (33)	26 (13)	104 (54)	193 (100)
Material B, patients, *n* (%)	52 (27)	28 (14)	116 (59)	196 (100)
Material C, patients, *n* (%)	20 (30)	10 (15)	37 (55)	67 (100)

*LNB* Lyme neuroborrelios

### Extraction of total nucleic acid and complementary DNA synthesis

For material A, total nucleic acid (NA) was extracted using the MagNa Pure LC Total Nucleic Acid Isolation Kit - Large Volume kit (Roche Applied Science, Penzberg, Germany) and BioRobot MagNa Pure LC 2.0 system (Roche Applied Science) according to the manufacturer´s instructions. Reverse-transcribed NA (RTNA) synthesis was performed using Illustra ™ Ready-to-Go RT-PCR beads kit (GE Healthcare, Amersham, Place, UK), previously described by Lager et al. 2017 [62]. A positive extraction control was included in each extraction containing sheep blood infected by *A. phagocytophilum* kindly provided by Prof Snorre Stuen, Department of Production Animal Clinical Sciences, Faculty of Veterinary Medicine, Norwegian University of Life Sciences, Norway. Since the same extracted material was used in the detection of all included pathogens, *A. phagocytophilum* alone was used as a control for the extraction. RNase-free (GE Healthcare) water was used as a negative extraction control. For material B, extraction of DNA from patient plasma was performed using Nucleic Acid Isolation Kit I (Roche Applied Science) in a MagNA Pure Compact Extraction Robot (Roche Applied Science), according to the manufacturer´s instruction. Regarding material C, DNA extraction of the CSF samples was performed using the NucliSENS easyMAG automated extraction platform (bioMérieux, Durham, NC, USA), following the manufacturer’s instructions. All sample materials used an insert sample volume of 400 µL and an elution volume of 50 µL.

### Detection of tick-borne pathogens by real-time PCR

In material A, four different TBPs, *A. phagocytophilum*, *N. mikurensis*,* Babesi*a spp. and *Rickettia* spp. were analysed using a CFX96™ real-time PCR detection system (Bio-Rad Laboratories, Inc., Hercules, CA). Primers for real-time PCR analysis were obtained from Invitrogen (Invitrogen, Carlsbad, CA) and probes from Life Technologies (Life Technologies, Carlsbad, CA). The primers and probes are presented in Supplementary Material 1. RNAse-free water (GE Healthcare) was used as a negative application control for all species.

### Anaplasma phagocytophilum

*A. phagocytophilum* detection was performed by a TaqMan real-time PCR reaction with primers and probe previously described by Henningsson et al. 2015 (Supplementary Material 1) [63]. The optimised PCR conditions in a final volume of 25 µL were: 12.5 µL TaqMan™ Fast Universal PCR Master Mix (2X), no AmpErase™ UNG (Thermo Fisher Scientific), 600 nM of each primer (Anaplasma-Fw and Anaplasma-Rv), 150 nM Anaplasma-probe, 5 µL template (cDNA), and RNase-free water up to 25 µL (GE Health Life Sciences). The following cycle conditions were used: 50 °C for 2 min, 95 °C for 10 min followed by 40 cycles of 95 °C for 15 s and 60 °C for 60 s. Positive control was the target sequence of the Anaplasma *gltA* gene (nucleotides 304–420) (Genebank: AF304137), which was synthesized and cloned into a vector pUC57 (Genscript USA Inc, NJ).

### Babesia species

*Babesia* spp. were detected by a SYBR Green real-time PCR detecting *18 S* rRNA with specific primers previously described by Casati et al. 2006 (Supplementary Material 1) [64]. The optimised PCR conditions in a final volume of 20 µL were: 10 µL Maxima SYBR Green qPCR Master Mix (2X), without ROX (Thermo Fisher Scientific), 200 nM of each primer (Babesia-Fw and Babesia-Rv), 5 µL template (cDNA), and RNase-free water (GE Healthcare Life Science) up to 20 µL. The cycling conditions were: 94 °C for 10 min, followed by 35 cycles of 94 °C for 1 min, 55 °C for 1 min, and 72 °C for 2 min, with a final extension of 72 °C for 5 min. The positive control constituted of a plasmid containing the target sequence of the gene *B. divergens 18 S* rRNA (nucleotides: 467–955) (GenBank: AJ439713), synthesized and cloned into a pUC57 vector (Genscript USA Inc, NJ).

### Neoehrlichia mikurensis

Two PCR methods were used for the detection of *N. mikurensis*: The method used in Material A consisted of a SYBR Green real-time PCR detecting *16 S* rRNA with specific primers previously described by Labbé Sandelin et al. 2015 (Supplementary Material 1) [65]. The optimised PCR conditions in a final volume of 20 µL were: 10 µL Maxima SYBR Green qPCR Master Mix (2X), without ROX (Thermo Fisher Scientific), 200 nM for each primer (NeoehrlichaA-Fw and 200 nM NeoehrlichaA-Rv), 5 µL template (cDNA), and RNase-free water (GE Healthcare Life Science) up to 20 µL. The cycling conditions were: 95 °C for 3 min, followed by 45 cycles of 95 °C for 15 s, 60 °C for 30 s, and 72 °C for 30 s. As a positive control, cDNA samples positive for *N. mikurensis* confirmed by sequencing in an earlier study from Labbé Sandelin et al. [65] were used.

The other method, used in Material B, was a Taqman real-time PCR directed against a 169-bp segment of the *groEL* gene of *N. mikurensis* previously described by Grankvist et al. [18]. 20 µL reaction mixture containing 1× FastStart Taqman Probe Master (Roche), 1 µM of each primer (NeoehrlichaB-Fw and NeoehrlichaB-Rv), 100 nM NeoehrlichiaB-probe, 4 µL DNA template, and RNase-free water (GE Healthcare Life Science) up to 20 µL (Supplementary Material 1). Real-time PCR analysis was performed at Rotorgene 6000 (QIAGEN, Hilden, Germany). Reaction conditions were 95 °C for 10 min, followed by 45 cycles at 95 °C for 15 s, and 54 °C for 1 min [18]. A synthetic plasmid containing the 169-bp sequence cloned into a pUC57 vector (Genscript) was used as a positive control.

### Rickettsia species

*Rickettsia* spp. in material A was detected by a TaqMan real-time PCR with specific primers and probe previously described by Stenos et al. 2005 (Supplementary Material 1) [66]. The optimised PCR conditions in a final volume of 20 µL were: 10 µL Maxima Probe qPCR Mix 2X (Thermo Fisher Scientific), 200 nM Rickettsia-Fw primer, 200 nM Rickettsia-Rv primer, 200 nM Rickettsia-probe, 5 µL template (cDNA), and RNase free water (GE Healthcare Life Science) up to 20 µL. The cycling conditions were: 50 °C for 3 min, 95 °C for 5 min, followed by 50 cycles of 95 °C for 20 s and 60 °C for 40 s. The positive control constituted a plasmid containing the target sequence of the *Rickettsia rickettsii gltA* gene (nucleotides 1102–1231) (GenBank: U59729), synthesized and cloned into a pUC57 vector (Genscript). For material C, the real-time PCR assay was performed according to Stenos et al. 2005 [66] using a Rotor-Gene 3000 (Corbett Research, Sydney, Australia) together with LC Taqman Master Kit (Roche Applied Science). The same sort of plasmid as for material A was used as positive control. All samples in material C were further analysed using a conventional nested PCR assay amplifying a 434-bp segment of the 17-kDa gene from *R. rickettsii*, as previously described by Carl et al. 1990 except for some minor changes (Supplementary Material 1) [67]. The PCR conditions were: 2.5 µL Qiagen PCR buffer (Qiagen), 500 nM Rickettsianest-Fw (TIB MOLBIOL Syntheselabor GmbH, Berlin, Germany), 500 nM Rickettsianest-Rv (TIB MOLBIOL), 5 µL Q-solution (Qiagen), 0.125 µL Taq polymerase (Qiagen), 0,5 µL dNTP, 5 µL DNA and RNAse free water (GE Healthcare Life Science) up until a final volume of 20 µL. The assay was performed in a GeneAmp^®^ PCR System 9700 (Applied Biosystems, Foster City, CA) with the following cycling: 40 cycles of 94 °C for 30 s, 57 °C for 2 min, and 70 °C for 2 min. A total of 10 µL obtained from this reaction was used as a template for the second PCR using the same reaction mixture volumes, primer concentrations, and PCR conditions as for the first PCR reaction.

## Results

**Table 3:** Results of real-time PCR analyses from all 235 patients in material A-C

	Plasma samples				CSF samples
	Anaplasma phagocytophilum	Babesia species	Neoehrlichia mikurensis	Rickettsia species	Rickettsia species
Material A, positive test/total number of samples	0/193	0/193	2^a^/193	0/193	NoT
Material B, positive test/total number of samples	NoT	NoT	0/196	NoT	NoT
Material C, positive test/total number of samples	NoT	NoT	NoT	NoT	0/67

*NoT* Not tested, *CSF* Cerebrospinal fluid

^a^ Initial positive test results could not be confirmed by further analysis using a species-specific real-time PCR detecting target gene (groEL)

In total, 235 children were analysed for potential eTBD. Among these 98 children had either a definite or a possible LNB and could thus potentially be presenting with another eTBD and/or a co-infection. Plasma samples analysed in material A were all negative for *A. phagocytophilum*, *Babesi*a spp., and *Rickettia* spp. by real-time PCR (Table 3).

However, two samples from two different patients were positive for *N. mikurensis* in the SYBR Green real-time PCR analysis (Table 3). The first patient was a 7-year-old boy with a previously recognised tick bite, presenting with fever, headache, neck stiffness, and loss of appetite for a week. Cerebrospinal fluid analysis at admission revealed pleocytosis with mononuclear dominance and positive *Borrelia*-specific antibody index for both IgG and IgM, thus fulfilling the criteria for definite LNB and a potential symptomatic co-infection along with *N. mikurensis.* The second patient was a 17-year-old boy with a previously recognised tick bite, presenting with acute facial nerve palsy, headache, and fatigue for a few days but with no fever. Analysis of CSF at admission showed no pleocytosis nor detectable *Borrelia-*specific antibodies. He was diagnosed with idiopathic facial nerve palsy and accordingly classified as non-LNB. This case could potentially be classified as an eTBD caused by *N. mikurensis.* However, none of the positive *N. mikurensis* samples could be confirmed by further analysis using a species-specific real-time PCR detecting target gene (*groEL*) performed at Sahlgrenska University Hospital, Gothenburg. Thus, in the end, these two samples were regarded as not determined.

The real-time PCR analyses on material B regarding *N. mikurensis* in plasma samples showed no positive results, nor did the real-time PCR analyses on material C regarding *Rickettsia* spp. in CSF (Table 3).

## Discussion

Ticks frequently act as vectors of multiple pathogens with the potential to cause disease if transmitted to humans via a tick bite. In our study, we did not find any molecular evidence for potential eTBDs (*A. phagocytophilum*, *N. mikurensis*,* Babesi*a spp., and *Rickettia* spp.) among Swedish children being evaluated for LNB, a patient group with potentially high exposure to TBPs. 

The clinical presentation of LNB in children is well described. In a review article from Bruinsma et al. 2023 [43], it was concluded that the most common neurological manifestation in children with LNB is acute facial nerve palsy and/or subacute meningitis, alongside other symptoms such as headache, fatigue, neck pain/stiffness, and fever. Less common symptoms are loss of appetite, nausea, and vertigo.

Human infections caused by tick-borne pathogens other than *B. burgdorferi* s.l. have, to our knowledge, not previously been thoroughly described, especially not in pediatric patients. Based on available data from case reports, case series, and studies on seroprevalences, eTBDs range from being asymptomatic to causing severe illness and may, in some cases, even have fatal outcomes. A strength of our study is the relatively large cohort consisting of 235 children of different ages (ranging from 1 to 19 years old), living in different areas in South-Central Sweden and with prospectively collected clinical data. Clinical symptoms documented within our study of children being evaluated for LNB are often unspecific and clearly overlap with symptoms of other potential eTBDs. In Europe, the most frequent and overlapping symptoms of eTBDs’ consist of fever, headache, myalgia and to some extent fatigue and malaise. However, infection caused by *A. phagocytophilum* may also present with prominent abdominal pain in the pediatric population [24, 25]. Rickettsiosis can cause symptoms of meningitis, and in rare cases even as isolated facial nerve palsy [32, 49]. Among patients with *Rickettsia* spp. infection, a maculopapular rash, or an eschar, which is a skin lesion caused by local vasculitis and necrosis, could also be seen. However, eschars are very unusual in infections caused by *R. helvetica* [32], which is the most common *Rickettsia* spp. among ticks in Sweden [68]. Babesiosis usually has a gradual onset of symptoms alongside with high intermittent fever, as well as loss of appetite [31]. *N. mikurensis* infections in immunocompromised patients may in addition to these overlapping symptoms also cause thromboembolic events and chills. However, in immunocompetent adult individuals, the clinical picture is more varied and may encompass no symptoms at all, to more severe manifestations such as sudden deafness, tinnitus and severe vasculitis. Importantly, to our knowledge, no cases of *N. mikurensis* in children in Sweden have been reported so far [35].

Emerging tick-borne diseases also present with altered laboratory findings, most commonly leukopenia (anaplasmosis and rickettsiosis), thrombocytopenia and elevated liver transaminases (anaplasmosis, rickettsiosis, and babesiosis), anaemia and reticulocytosis (babesiosis) and anaemia, leucocytosis and elevated levels of both C-Reactive Protein (CRP) and orosomucoid (neoehrlichiosis) [24, 25, 31–35]. Unfortunately, no data on blood cell count, transaminases or CRP were available in our present study.

Although patients in our cohort presented with clinical manifestations potentially matching several different eTBDs, no laboratory evidence of infection, based on PCR, could be found. Similar results were published in a previous study conducted by Gyllemark et al. 2021 [69]. In that study potential central nervous system infections caused by various TBPs were investigated by using molecular and serological testing to evaluate whether clinicians overlook eTBDs in patients being investigated for LNB [69]. Serum and CSF samples from 600 patients, both children and adults, were retrospectively analysed. Although real-time PCR analysis detected *N. mikurensis* DNA in one serum sample, using two PCR methods targeting different genes for confirmation (*16 S rRNA* and *groEL*) [18], the authors concluded that the risk of contracting a symptomatic infection with these various TBPs appeared to be low in the current setting. In our study, we had two *N. mikurensis*-positive samples. However, the two samples were only positive in the real-time PCR using SYBRGreen, a less specific PCR, and the findings could not be confirmed by the probe-based real-time PCR targeting the *groEL* gene performed at Sahlgrenska University Hospital (which is the Swedish reference laboratory for *N. mikurensis*). Thus, results were thereby considered as not determined.

Ticks carrying more than one pathogen at the same time appear to be quite common [52, 55]. This study wasn´t able to detect any eTBD in addition to a potential LNB diagnosis. Consequently, it wasn´t possible to evaluate potential tick-borne co-infections/co-diseases. Boyer et al. [56] conclude that human tick-borne co-diseases are rare but do occur. However, there is still a current gap of knowledge regarding both the frequency and the severity of tick-borne co-infections/co-diseases.

Henningsson et al. [30] systematically reviewed laboratory methods for detection of TBPs in humans and highlighted that, outside LB and TBE, there is a lack of high-quality evidence studies. Moreover, the method of choice can be dependent the phase of the disease and a combination of different methodologies may be required to achieve higher sensitivity. Unfortunately, this approach was not possible in our study due to limited volumes of samples. 

We primarily used real-time PCR in our study, as positive results more clearly indicate the presence of infection with a specific pathogen, and, together with clinical manifestations, better answer the question of potentially overlooked infections in the clinical setting. However, the sensitivity of real-time PCR can vary between different pathogens and the stage of illness [30], and there is a a risk of missing an eTBD. Serological analyses, particularly with acute and convalescent samples, could have given complementary information about these TBPs within the clinical setting. However, the available sample volume was insufficient to perform both tests, and for material A-B, there were no follow-up samples. Another study limitation is the lack of information from other complementary laboratory analyses such as blood cell count, liver transaminases and CRP. Although they could have been helpful in the clinical setting regarding eTBDs, they were not part of the standardized diagnostic workup regarding LNB in children and thus these parameters are missing.

Our study found no evidence for infection with *Anaplasma phagocytophilum*, *Neoehrlichia mikurensis*, *Babesia *spp and *Rickettsia *spp in children with Lyme neuroborreliosis in Sweden from 2011 to 2014. It is possible that 10-15 years later the situation may look different, and that new eTBP may have emerged. Neither *Borrelia miyamotoi *nor *Spiroplasma ixodetis* were included in this study but may be considered in future studies regarding eTBDs *Borrelia miyamotoi* nor *Spiroplasma ixodetis* [70, 71] as well as the use of additional analysing modalities, particularly serology.

## Conclusion

We conclude that molecular evidence for potential eTBDs was not found within a cohort of Swedish pediatric patient with potential high exposure to tick-borne pathogens. However, given the indications of the emergence of ticks and several tick-borne pathogens, we suggest that eTBDs should be considered as differential diagnoses in patients with non-LNB, atypical LNB or unexplained fever and skin rashes, including a complete blood count and liver enzymes in the diagnostic workup.

## Supplementary Information

Supplementary Material 1.

## Data Availability

The datasets used and/or analysed during the current study are available from the corresponding author on reasonable request.

## Abbreviations

eTBD: emerging tick-borne diseases
TBP: tick-borne pathogens
LNB: lyme neuroborreliosis
spp.: species
PCR: polymerase chain reaction
LB: lyme borreliosis
s.l.: sensu lato
TBE: tick-borne encephalitis
CSF: cerebrospinal fluid
NA: nucleic acid
CRP: C-Reactive Protein
AI: antibody index
n: number
Ig: immunoglobulin
NoA: not available
bp: base pair
C: celcius
nM: nano mole
NoT: not tested
