# Supplementary material for: Are there other undiagnosed tick-borne infections in children being evaluated for Lyme neuroborreliosis?
Source: BMC Pediatr. 2026 Jun 15;26:565. doi: 10.1186/s12887-026-07000-4 (PMC13274043; doi:10.1186/s12887-026-07000-4)
Supplement: Supplementary file 1 — Supplementary Material 1. [file 12887_2026_7000_MOESM1_ESM.pdf]

Supplementary Material 1: Used primer and probes including primer and probe sequence, target gene, product length, concentration and reference for each species.

Agents	Primer name	Sequence (5´→ 3´)	Function	Target gene	Length (bp)	Concentration (nM)	References
Anaplasma phagocytophilum	Anaplasma-Fw	TTTTGGGCGCTGAATACGAT	Forward	*gltA*	64	600	Henningsson et al. 2015 (63)
Material A	Anaplasma-Rv	TCTCGAGGGAATGATCTAATAACGT	Reverse			600	
	Anaplasma-probe	VIC-TGCCTGAACAAGTTATG-MGBNFQ	Probe			150	
***Babesi*a species**	Babesia-Fw	GTCTTGTAATTGGAATGATGG	Forward	*18S* rRNA	411-452[a]	200	Casati et al. 2006 (64)
Material A	Babesia-Rv	TAGTTTATGGTTAGGACTACG	Reverse			200	
Neoehrlichia mikurensis	NeoehrlichiaA-Fw	GTAAAGGGCATGTAGGCGGTTTAA	Forward	*16S* rRNA	107	200	Labbé Sandelin et al. 2015 (65)
Material A	NeoehrlichiaA-Rv	TCCACTATCCTCTCTCGATCTCTAGTTTAA	Reverse			200	
Material B	NeoehrlichiaB-Fw	CGGAAATAACAAAAGATGGA	Forward	groEL	169	1000	Grankvist et al. 2015 (18)
	NeoehrlichiaB-Rv	ACCTCCTCGATTACTTTAG	Reverse			1000	
	NeoehrlichiaB-probe	6-FAM-TTGGTGATGGAACTACA-MGB	Probe			100	
***Rickettsia* species**	Rickettsia-Fw	TCGCAAATGTTCACGGTACTTT	Forward	*gltA*	74	200	Stenos et al. 2005 (66)
Material A and C	Rickettsia-Rv	TCGTGCATTTCTTTCCATTGTG	Reverse			200	
	Rickettsia-probe	6-FAM-TGCAATAGCAAGAACCGTAGGCTGGATG-MGBNFQ	Probe			200	
Material C	Rickettsianest-Fw	GCTCTTGCAACTTCTATGTT	Forward	17kDa	434	500	Carl et al. (67)
	Rickettsianest-Rv	CATTGTTCGTCAGGTTGGCG	Reverse			500	

[a] Depending on the *Babesia* species.

bp: Base pair

C: Celsius

nM: Nano mole
